# Metabolic interplay between cytosolic phospho*enol*pyruvate carboxylase and mitochondrial alternative oxidase in thermogenic skunk cabbage, *Symplocarpus renifolius*

**DOI:** 10.1080/15592324.2016.1247138

**Published:** 2016-10-14

**Authors:** Md. Abu Sayed, Yui Umekawa, Kikukatsu Ito

**Affiliations:** a United Graduate School of Agricultural Science, Iwate University, Ueda, Morioka, Iwate, Japan; b Cryobiofrontier Research Center, Faculty of Agriculture, Iwate University, Ueda, Morioka, Iwate, Japan

**Keywords:** Alternative oxidase, mitochondria, phospho*enol*pyruvate carboxylase, respiration, skunk cabbage, thermogenesis

## Abstract

Skunk cabbage (*Symplocarpus renifolius*) blooms in early spring and its inflorescence, referred to as the spadix, can produce enough heat to melt snow. Here, we investigated glycolytic carbon flow at the PEP branch-point in thermogenic spadices. Our analyses revealed that petals and pistils in thermogenic florets exhibited higher expression of *SrPEPC* and *SrAOX* transcripts than those of *SrPK, SrPEPCK*, and *SrPEPtase*. Moreover, enzymatic analyses showed high activities of PEPC in the extracts from thermogenic florets. Finally, mitochondria from thermogenic florets showed low respiratory activities when pyruvate was used as a substrate, although a significant malate-mediated cyanide-insensitive respiration was observed. Collectively, these results suggest that PEP metabolism, primarily catabolized by PEPC, plays a critical role in thermogenesis in *S. renifolius*.

## Abbreviations

AOX: alternative oxidase
BSA: bovine serum albumin
EDTA: ethylenediaminetetraacetic acid
EF1α: elongation factor 1α
MOPS: 3-morpholinopropanesulfonic acid
MPCs: mitochondrial pyruvate carriers
NADH: nicotinamide adenine dinucleotide
PCR: polymerase chain reaction
PEP: phospho*enol*pyruvate
PEPC: phospho*enol*pyruvate carboxylase
PEPCK: phospho*enol*pyruvate carboxykinase
PEPtase: PEP phosphatase
PK: pyruvate kinase
qRT-PCR: quantitative real-time PCR
TPP: thiamine pyrophosphate

## Introduction

Glycolysis is a central pathway of carbohydrate metabolism among living organisms from bacteria to plants and humans.^1,2,3^ In the glycolytic pathway, phospho*enol*pyruvate (PEP) is an important intermediate, as it occupies the highest position on the thermodynamic scale of known phosphorylated metabolites.^4^ In animal cells, PEP is predominantly catalyzed by pyruvate kinase (PK; EC 2.7.1.40) with the concomitant phosphorylation of ADP to ATP.^3^ However, in addition to PK, PEP carboxylase (PEPC; EC 4.1.1.31) and PEP phosphatase (PEPtase; EC 3.1.3.60) play roles in the catabolism of PEP and overall regulation of mitochondrial respiration in plant cells.^2,5^ In contrast, PEP carboxykinase (PEPCK; EC 4.1.1.49) is an enzyme involved in gluconeogenesis.^3^

Although thermogenesis is uncommon in plants, the glycolytic pathway is important in thermogenic plants such as skunk cabbage (*Symplocarpus renifolius*), which utilize carbohydrates as a major respiratory substrate for their metabolic heat-production.^6^ In the case of *S. renifolius*, organ-specific thermogenesis occurs in the inflorescence known as the spadix, and maintains a temperature of approximately 23°C during flowering, even when ambient temperatures drop below freezing.^7,8,9^ Because the mitochondrial cyanide-insensitive alternative oxidase (AOX) allows for a dramatic decrease in free energy between ubiquinol and oxygen,^10,11^ *SrAOX* identified in *S. renifolius* is likely to play a role in thermogenesis in this plant.^7,12,13^ In our previous study, sustained thermogenesis in spadices was associated with the import of carbohydrates including sucrose, glucose, and fructose from roots.^14^ Although these results suggest that glycolysis and subsequent AOX-mediated mitochondrial respiration plays a crucial role in organ-specific thermogenesis in *S. renifolius*, the mechanisms of carbohydrate metabolism in this plant remain poorly understood.

The purpose of the present study was to investigate glycolytic carbon flow at the PEP branch-point in thermogenic spadices of *S. renifolius*. Our data showed that co-expression of *PEPC* and *AOX* is central for metabolic heat-production in this plant.

## Results

### Gene expression analyses for PK, PEPtase, PEPC, and PEPCK in thermogenic and non-thermogenic tissues

**Figure 1.**
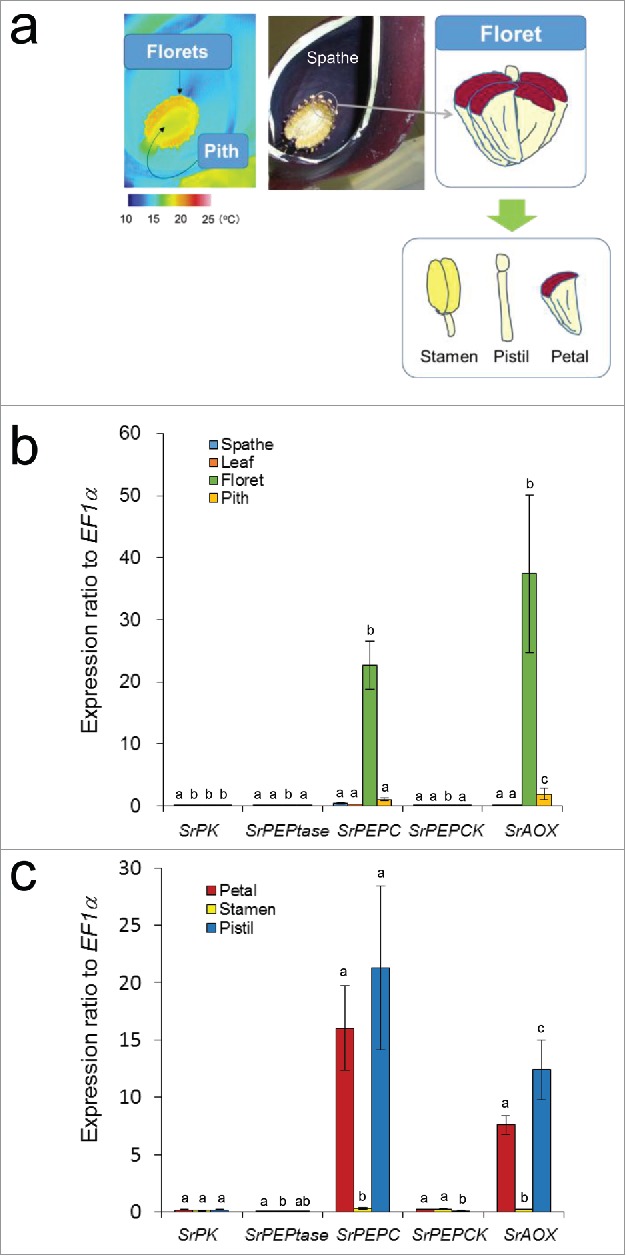
Levels of *SrPK, SrPEPtase, SrPEPC, SrPEPCK*, and *SrAOX* transcripts in various tissues during thermogenesis in *S. renifolius*. (a) Infrared thermal imaging of longitudinal section of the spadix. The positions of florets, pith, and spathe are shown. Each floret composed of the stamen, pistil, and petal is also illustrated. Expression profiles of *SrPK, SrPEPtase, SrPEPC, SrPEPCK*, and *SrAOX* transcripts in the (b) spathe, leaf, floret, and pith and (c) petal, stamen, and pistil. *SrEF1*α transcripts were used as a normalization control. Experiments were performed in triplicate for each sample. Data are expressed as the mean ± standard deviation. Values with different letters in the graph indicate that they are statistically significantly different (*n* = 3; *P* < 0.05). AOX, alternative oxidase; PK, pyruvate kinase; PEP, phospho*enol*pyruvate; PEPtase, PEP phosphatase; PEPC, PEP carboxylase; PEPCK, PEP carboxykinase.

Tissue-specific expression patterns of *SrPK, SrPEPtase, SrPEPC*, and *SrPEPCK* transcripts were determined by qRT-PCR using RNAs from the spathe, leaf, floret, and pith tissues collected from *S. renifolius* plants during thermogenesis (Fig. 1). In these experiments, temperatures of ambient air and thermogenic spadix were 12.2°C and 22.3°C, respectively. Our previous study showed that florets are thermogenic, while the spathe, leaf, and pith are non-thermogenic.^7^ Thermogenic florets contain the stamen, pistil, and petal (Fig. 1a). *SrAOX* mRNA expression was used as a thermogenic tissue-specific control to reflect the thermogenic status of the samples. Our data clearly showed that expression levels of the *SrPEPC* and *SrAOX* transcripts were significantly higher in florets than those in the non-thermogenic spathe, leaf, and pith tissues (Fig. 1b). In contrast, the expression levels of *SrPK, SrPEPtase*, and *SrPEPCK* were nearly undetectable in the spathe, leaf, floret, and pith tissues (Fig. 1b).

Next, to examine gene expression in more detail, we focused on 3 tissues, the petal, stamen, and pistil, each of which comprises the florets of the spadix (Fig. 1a). *SrPEPC* transcripts were again co-expressed with *SrAOX* transcripts in petals and pistils, whereas *SrPK, SrPEPtase*, and *SrPEPCK* transcripts were expressed at low levels in all tissues examined (Fig. 1c). Expression levels of *SrPK, SrPEPtase, SrPEPC, SrPEPCK*, and *SrAOX* were also determined at 2 thermogenic stages of plants collected on the same day, and showed considerably higher expression of *SrPEPC* and *SrAOX* transcripts in the floret, petal, and pistil tissues (Figs. S1 and S2).

### Enzyme assays for PK, PEPtase, PEPC, and PEPCK

To determine whether similar high enzymatic activities of PEPC are found in thermogenic florets, we determined the PK, PEPtase, PEPC, and PEPCK activities in cytosolic fractions from thermogenic florets. These analyses showed that the specific activity of PEPC was significantly higher than that of PK, PEPtase, and PEPCK (Table 1). Another independent preparation from different thermogenic plants also showed similar results (Supplementary Table 1).

**Table 1.** Enzyme activities of PK, PEPtase, PEPC, and PEPCK in cytosolic fraction of thermogenic florets of *S. renifolius*.

Enzyme	Specific activity (nmol min^−1^mg^−1^protein)
PK	9.5 ± 2.1^a^
PEPtase	10.2 ± 4.3^a^
PEPC	107.5 ± 13.7^b^
PEPCK	14.8 ± 5.7^a^

Value for PEPCK is depicted as decarboxylation activity. Assays were performed in triplicate for each sample. Data are expressed as mean ± standard deviations. Values with different letters indicate that they are statistically significantly different (*n* = 3; *P* < 0.05). PK, pyruvate kinase; PEP, phospho*enol*pyruvate; PEPtase, PEP phosphatase; PEPC, PEP carboxylase; PEPCK, PEP carboxykinase.

### Predicted amino acid sequence of SrPEPC

**Figure 2.**
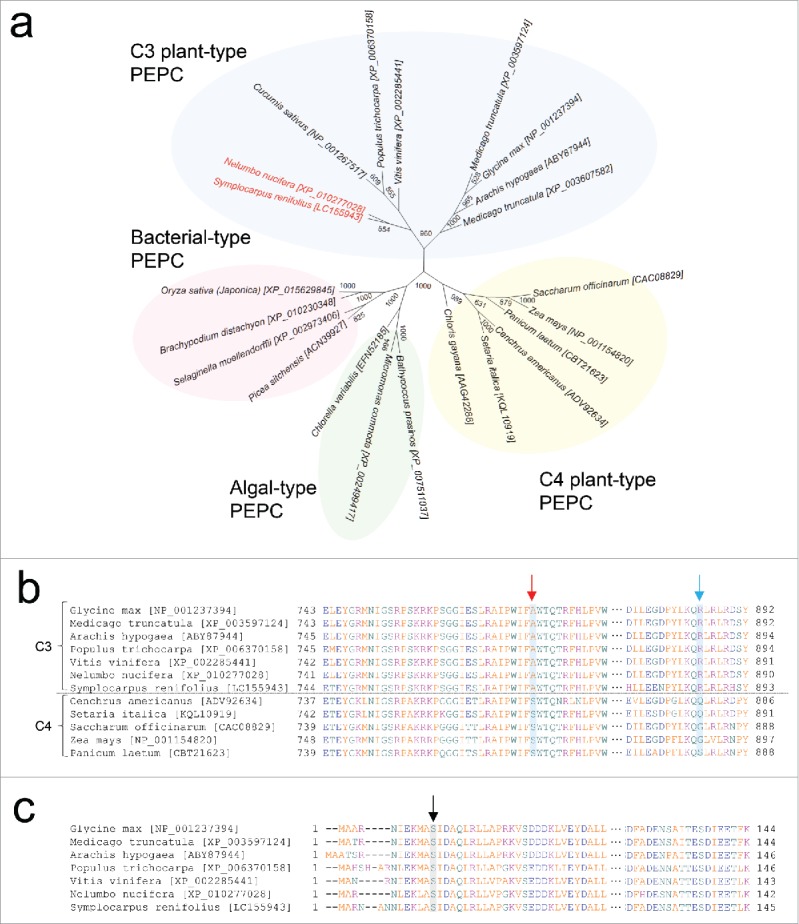
Phylogenetic tree and amino acid alignments of PEPC proteins. (a) Phylogenetic tree of deduced amino acid sequences of PEPC from *S. renifolius*, other C3- and C4-plants, and bacterial and algal type organisms. (b) Positions of HCO_3_^−^-binding loop (red arrow) and inhibitor binding site (blue arrow). (c) Conserved potential phosphorylation sites (black arrow) at the N-terminal region of PEPC. PEPC, phospho*enol*pyruvate carboxylase.

Phylogenetic analysis of PEPC amino acid sequences from 15 plants, 3 algae, and 4 bacteria clearly characterized SrPEPC as a C3-plant-type PEPC (Fig. 2a). Moreover, amino acid sequences of SrPEPC and the homolog NnPEPC from the thermogenic plant species *Nelumbo nucifera*^15^ were closely related on the phylogenetic tree (Fig. 2a).

Multiple sequence alignments of the deduced PEPC amino acid sequence with other protein sequences from C3- and C4-plant PEPCs showed that SrPEPC possesses conserved alanine and arginine residues, characteristic of C3-plant-type PEPCs^16^ (Fig. 2b). Moreover, a serine residue with a potential phosphorylation site^5^ was highly conserved across C3- and C4-types of plant PEPCs, including that from *S. renifolius*.

### Substrate-dependent oxygen uptake

**Figure 3.**
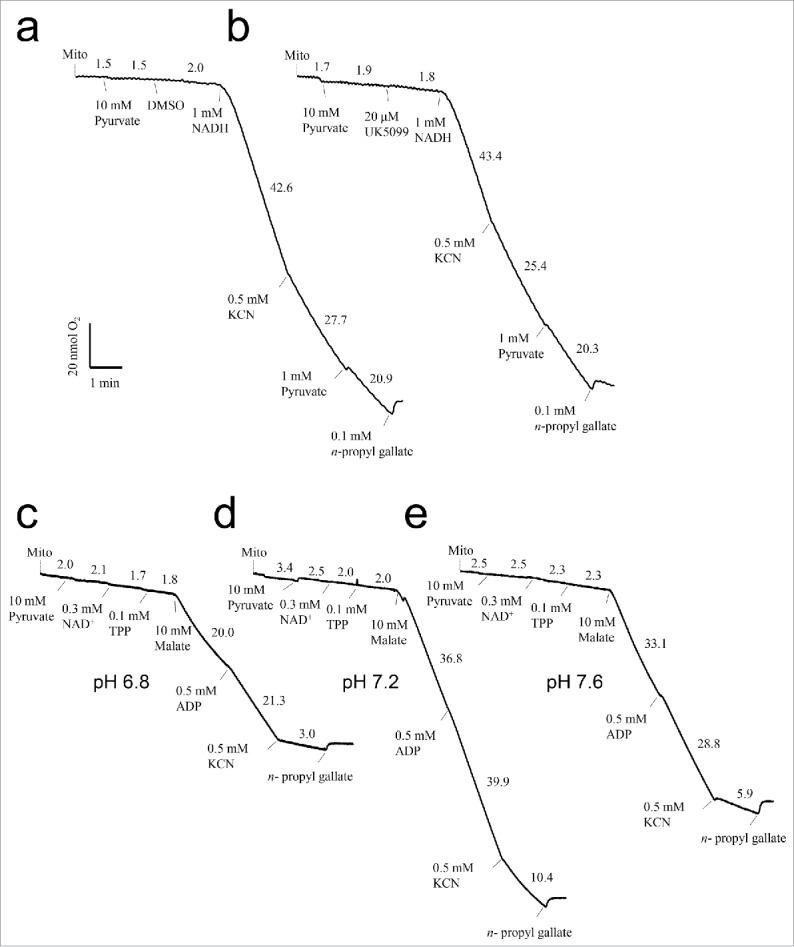
Oxygen consumption of intact mitochondria purified from thermogenic florets of *S. renifolius*. Substrates and compounds were added using freshly prepared mitochondria. Values over the traces indicate oxygen consumption in nmol of O_2_ per min. (a, b) Mitochondrial respiration rates with pyruvate as a substrate in a buffer without cofactors at pH 7.2^23^ (c–e). Mitochondrial respiration rates with pyruvate as a substrate in the presence of cofactors NAD^+^ and TPP at pH 6.8 (c), 7.2 (d), and 7.6 (e). All assays were performed at 25°C. Typical results are shown, representing 3 independent respiration assays. NAD, nicotinamide adenine; TPP, thiamine pyrophosphate.

The present results indicate that pyruvate, which is formed from PEP by PK and/or PEPtase, is not a major end-product of the glycolytic pathway in thermogenic cells of *S. renifolius*. Thus, we next wished to clarify whether purified mitochondria from thermogenic florets oxidize pyruvate as a substrate for respiration. In this analysis, we used the same experimental conditions at pH 7.2 as previously reported for pyruvate-mediated respiration in the mitochondria from thermogenic appendices of *Arum maculatum*^17^ (Fig. 3a and b). In these experiments, we used the cinnamate derivative UK5099 to inhibit mitochondrial pyruvate transport^17,18^ and determined the effects on respiratory rates (Fig. 3b). Our results showed that pyruvate does not act as a substrate for mitochondrial respiration and that UK5099 does not affect respiration. However, subsequent addition of NADH led to significantly increased respiration rates both in absence and in presence of cyanide, indicating cyanide-insensitive mitochondrial AOX activities (Fig. 3a and b).

To confirm that our purified mitochondria from thermogenic florets do not utilize externally added pyruvate as a substrate for respiration, we performed experiments at pH 6.8, 7.2, and 7.6 in presence of the pyruvate dehydrogenase complex cofactors NAD^+^ and thiamine pyrophosphate (TPP).^19^ These experiments indicated that pyruvate was not used as a respiratory substrate (Fig. 3c–e). However, addition of exogenous malate led to increased respiration rates regardless of whether cyanide was present, with the highest respiration rate observed at pH 7.2 (Fig. 3c–e).

## Discussion

PEP is an energy-rich compound and its metabolism in replacement of phosphate group plays an important role in glycolysis.^4,5^ Therefore, analysis of the metabolic partitioning of PEP will provide insight into the glycolytic end products and energy substrates used in subsequent mitochondrial respiration.

Herein, we showed that transcripts encoding the C3-type of PEPC protein SrPEPC were specifically and highly co-expressed with those encoding SrAOX in the petals and pistils of thermogenic florets, whereas the expression levels of *SrPK, SrPEPtase*, and *SrPEPCK* transcripts were extremely low in all tissues examined (Fig. 1). Moreover, enzymatic activities of PEPC in thermogenic florets were higher than those of PK, PEPtase, and PEPCK (Table 1), indicating that PEP is predominantly catabolized by PEPC in thermogenic tissues of *S. renifolius*. Although extremely high enzymatic activities of PEPC have been reported in other thermogenic plants including *A. maculatum*,^20^ this is the first study to show tissue-specific co-expression of *PEPC* and *AOX* in quantitative gene expression analyses of thermogenic plants.

**Figure 4.**
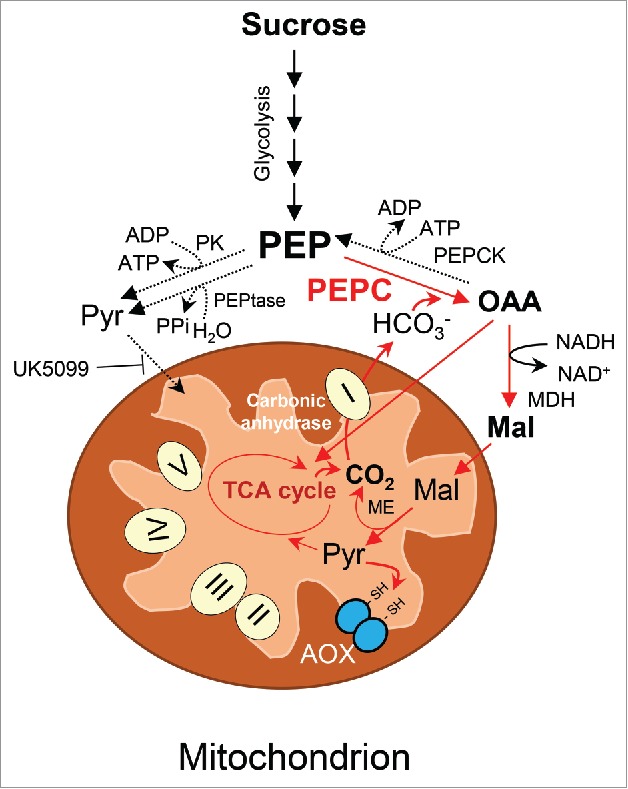
PEP-mediated metabolism in thermogenic cells of *S. renifolius*. PEP yielded by glycolytic pathway is predominantly catabolized by PEPC to produce oxaloacetic acid (OAA). OAA is either directly oxidized in the mitochondria or converted to malate (Mal) by cytosolic malate dehydrogenase (MDH). Malate is catabolized by the malic enzyme (ME) in mitochondria to produce pyruvate (Pyr) and carbon dioxide (CO_2_). Intra-mitochondrially produced pyruvate can either enter the TCA cycle or allosterically activate the alternative oxidase (AOX). The CO_2_ produced in the TCA cycle and by the malic enzyme is catabolized by complex I-integrated mitochondrial carbonic anhydrase to form bicarbonate (HCO_3_^−^) and is catabolized by PEPC to yield OAA. Expression of PEPC and AOX can enhance the recycling of respired CO_2_ and energy dissipative mitochondrial respiration. Major metabolic pathways predicted in the present study are depicted by red arrows. Complexes I–V of the electron transport chain are shown. UK5099, an inhibitor of the pyruvate transporter, is also depicted. PK, pyruvate kinase; PEP, phospho*enol*pyruvate; PEPCK, phospho*enol*pyruvate carboxykinase (PEPCK); PEP phosphatase (PEPtase); PPi, pyrophosphate; Suc, succinate; Cit, citrate.

Because the AOX-mediated energy-dissipative respiration pathway contributes significantly to cellular thermogenesis in plants, co-expression of *SrAOX* and *SrPEPC* detected in the present study may be critical for metabolic cross-talk between the cytosol and AOX-expressing mitochondria in thermogenic cells (Fig. 4). In thermogenic cells, PEP was primarily catabolized by PEPC to produce oxaloacetic acid, which is used directly as a mitochondrial respiration substrate or is converted to malate by malate dehydrogenase^21,22^ for use as a respiratory substrate (Fig. 3c–e). In either case, such PEPC-mediated metabolism may contribute significantly to continuous carbon flow in furnishing C4-dicarboxylic acids that maintains increased respiration for thermogenesis in *S. renifolius*. These data are consistent with those of previous report of constitutive PEPC-overexpressing transgenic plants, in which carbon flow was redirected from soluble sugars to organic acids.^23^ More importantly, because PEPC catalyzes the addition of HCO_3_^−^ to PEP,^4^ excess CO_2_ that is liberated with increased mitochondrial respiration in thermogenic cells may be catabolized by complex I-integrated mitochondrial γ-carbonic anhydrases to form HCO_3_^−^,^24,25^ which is subsequently converted to oxaloacetic acid by PEPC. Previously, it was shown that PEPC is highly expressed and participates in the recycling of respired CO_2_ in the spikelets of C3-type plants.^26^ Similarly, fruiting plants possess a system known as fruit photosynthesis,^27^ in which CO_2_ from mitochondrial respiration is refixed by PEPC. These data indicate that thermogenic plants express higher levels of PEPC and AOX enzymes in their non-photosynthetic organs, such as in the spadices of *S. renifolius* which developed specialized metabolisms not only for recycling of excess CO_2_ similar to that seen in other C3 plants but also for energy-dissipating AOX-mediated mitochondrial respiration during evolution. Accordingly, integration of PEPC-mediated CO_2_ assimilation and AOX-mediated mitochondrial respiration probably act as substantial carbon resources in thermogenic plants.

In the present study, mitochondria purified from thermogenic florets of *S. renifolius* did not oxidize exogenous pyruvate as a respiratory substrate in the presence or absence of cofactors (Fig. 3). In contrast, mitochondria from thermogenic spadices of *A. maculatum* were previously shown to oxidize exogenous pyruvate in the absence of exogenous cofactors.^18^ In addition, rapid oxidation of pyruvate by isolated *A. maculatum* mitochondria was sensitive to UK5099, suggesting the presence of mitochondrial pyruvate carriers, as shown recently in *Arabidopsis*.^28^ Hence, expression of mitochondrial pyruvate carriers in thermogenic florets of *S. renifolius* may be lower than in those of *A. maculatum*. Because pyruvate has been identified as an allosteric activator of *S. renifolius* AOX,^12^ AOX activities in this plant may be post-translationally regulated by intra-mitochondrially produced pyruvate via malic enzyme^29,30^ that uses malate from the PEPC-mediated metabolic pathway. It should be noted here that NAD-dependent malic enzyme α- and/or β-subunits have been identified in mitochondria from thermogenic florets of *S. renifolius*.^13^

In conclusion, we report that *SrPEPC* is abundantly co-expressed with *SrAOX* in thermogenic florets of *S. renifolius*. These results further suggest that PEPC plays a role in metabolic heat-production in furnishing C4-dicarboxylic acids to AOX-expressing mitochondria in other thermogenic plants.

## Materials and methods

### Plant materials

All plant materials were sampled from wild *S. renifolius* grown outdoors. For thermal imaging, *S. renifolius* plants that were transplanted from Hakuba (Nagano prefecture, Japan) to Iwate University campus (Iwate prefecture, Japan) in April 2005 were used. For preparation of total RNAs, fresh spathe, leaf, floret and pith were collected from *S. renifolius* at Fujine (Iwate prefecture, Japan) on April 3, 2012 and at Omori (Akita prefecture, Japan) on April 1, 2014. Mitochondria were purified from spadices of *S. renifolius* sampled at Omori on March 27, 2015. For enzyme assays, florets were collected from thermogenic spadices of *S. renifolius* that were sampled at Omori on April 20, 2016.

### Thermal imaging and temperature measurements

Thermal images were obtained using an infrared thermal camera as described previously.^7^ Temperatures of the spadices were measured using an automatic recording thermometer (TR-52; T & D, Nagano, Japan).

### Total RNA extraction, cDNA amplification, and isolation of full-length cDNAs encoding SrPK, SrPEPtase, SrPEPC, and SrPEPCK

Total RNAs were extracted using either an RNeasy Plant Mini Kit (Qiagen, Hilden, Germany) or a FastPure® RNA Kit (Takara Bio, Shiga, Japan). First-strand cDNA synthesis was performed using PrimeScript™ II 1st strand cDNA Synthesis Kit (Takara Bio) with oligo-dT primers provided by the manufacturer. Procedures for cDNA cloning are described in the Supplementary information. Briefly, partial fragments of targeted genes for *SrPK, SrPEPtase, SrPEPC*, and *SrPEPCK* were first amplified using PCR with *Takara Ex Taq*® (Takara Bio) and the primers are listed in Supplementary Table 2. Gene-specific primers were then designed to perform 5′- and 3′-rapid amplification of cDNA ends using SMARTer™RACE cDNA Amplification Kit (Clontech, Mountain View, CA, USA). To isolate full-length cDNAs, final PCR amplifications were performed with the KOD -Plus- (Toyobo, Osaka, Japan) with the primers shown in Supplementary Table 2. The obtained fragments were cloned into the T-Vector (pMD19, Takara Bio) and sequenced in both directions. Full-length sequences were determined by isolating at least 2 independent clones with identical sequences. DNA sequences were analyzed using GENETYX software (Genetyx, Tokyo, Japan). Complete cDNAs encoding *SrPEPC, SrPK, SrPEPCK*, and *SrPEPtase* were deposited in the DNA Data Bank of Japan with Accession numbers LC155943, LC155944, LC155945, and LC155946, respectively.

### Expression analyses of genes encoding SrPK, SrPEPtase, SrPEPC, and SrPEPCK

Real-time qPCR was performed using a Thermal Cycler Dice (TP800; Takara Bio) instrument as described previously.^13,31^ Gene-specific primers (Supplementary Table 2) were designed from identified cDNAs and the housekeeping gene *EF1*α was used as a normalization control.^31^

### Phylogenetic tree analyses of PEPC proteins and sequence alignments

A phylogenetic tree was constructed using the neighbor-joining method with ClustalW^32^ for 22 PEPC proteins gathered from GeneBank®. A bootstrap consensus tree was inferred from 1000 replicates to represent the evolutionary history of the present taxa. Branches corresponding to partitions that were reproduced in less than 50% of bootstrap replicates were collapsed. Multiple sequence alignments were performed using GENETYX software.

### Enzyme assays

Enzyme activities of PK, PEPtase, PEPC, and PEPCK were determined in thermogenic florets after extraction in ice-cold extraction buffer containing 0.3 M mannitol, 20 mM MOPS (pH 7.5), 2 mM EDTA, 2 mM pyruvate, 7 mM cysteine, and 0.2% BSA. Extracts were filtered through 8 layers of Miracloth (EMD Millipore, Billerica, MA, USA). Filtrates were collected in 50-mL tubes and centrifuged at 120 g for 10 min at 4°C. Supernatants were collected and centrifuged again at 12,000 g for 10 min at 4°C, and stored at −80°C until enzymatic analyses. Enzyme assays of PK and PEPtase and assays of PEPC and PEPCK for decarboxylation were conducted as previously described^33,34,35^ with a double beam spectrophotometer (Biospec-1600, Shimadzu, Kyoto, Japan) at 25°C.

### Isolation of intact mitochondria and respiration analyses

Mitochondria were isolated from *S. renifolius* spadices as described previously.^36^ Oxygen uptake by mitochondria was then measured according to our previous reports^31,36^ at 25°C.

### Determination of protein concentrations

Protein concentrations of isolated mitochondria and crude extracts were determined as described previously.^15^

## Statistical analysis

All data were compared using one-way factorial ANOVA (SigmaPlot 12, Systat Software, San Jose, CA, USA). Tukey's honest significance posthoc tests were used to identify significantly different means. Significant differences between means were calculated at *P* = 0.05.

## Supplementary Material

Supplemental_data.zip
