# Supplementary material for: Metabolic interplay between cytosolic phosphoenolpyruvate carboxylase and mitochondrial alternative oxidase in thermogenic skunk cabbage, Symplocarpus renifolius
Source: Plant Signal Behav. 2016 Oct 14;11(11):e1247138. doi: 10.1080/15592324.2016.1247138 (PMC5157899; doi:10.1080/15592324.2016.1247138)
Supplement: Supplemental_data.zip [file kpsb-11-11-1247138-s001.zip › Supplemental data/1. Supplementary information_Final.docx]

**Supplementary information**

**Procedures for cDNA cloning of *PK*, *PEPtase*, *PEPC*, and *PEPCK***

Partial fragments of genes encoding *PK*, *PEPtase*, *PEPC*, and *PEPCK* were initially PCR amplified using first strand cDNA and Taq DNA polymerase (*Takara Ex Taq^®^*, Takara Bio). Primers were designed to target conserved *PK*, *PEPtase*, *PEPC*, and *PEPCK* gene sequences shared by *Arabidopsis thaliana*, *Oryza sativa*, *Solanum tuberosum*, *Zea mays*, and *Musa acuminata subsp. Malaccensis*, and are denoted PKF1-PKR1, PEPtaseF1-PEPtaseR1, PEPCF1-PEPCR1, and PEPCKF1-PEPCKR1 (Supplementary table 1), respectively. PCR amplification (My Cycler^TM^ Thermal Cycler, BioRAD) was performed under the following conditions: 1 cycle at 94°C for 1 min, followed by 35 cycles of 94°C for 30 s, 50°C (for *PEPC* and *PEPCK*) or 58°C (for *PK*) for 30 s, and 72°C for 30 s, with a final extension at 72°C for 7 min. PCR amplification of *PEPtase* was performed under the following conditions: 1 cycle at 94°C for 2 min, followed by 35 cycles of 94°C for 30 s, 50°C for 30 s and 68°C for 2 min with a final extension at 68°C for 5 min. PCR products were electrophoresed in 1% agarose gels and DNA bands of expected sizes were excised and purified using Nucleospin^®^ Gel and PCR Clean-Up (Takara Bio). Purified DNA fragments were then ligated into T-Vector (pMD19, Takara Bio, or pCR2.1, Thermo Fisher Scientific) using DNA Ligation Kit <Mighty Mix> (Takara Bio) and were transfected into *E.coli* cells. Expected colonies expressing *PK*, *PEPtase*, *PEPC*, and *PEPCK* were identified and plasmids were purified by LaboPass^TM^ Mini Plasmid DNA Purification Kit (Hokkaido System Science) for sequencing. Gene specific primers were then designed for 5ʹ- and 3ʹ-Rapid Amplification of cDNA Ends (RACE) using SMARTer^TM^ RACE cDNA Amplification Kit (Takara Bio). SrPKF2, SrPKF3, SrPKF4 and SrPKR2, SrPKR3, SrPKR4 (Sr indicates *Symplocarpus renifolius*) were used to amplify targeted genes with Universal Primer Mix (UPM) for 3ʹ- and 5ʹ-RACE of *SrPK*, respectively. PCR amplification was performed under the following conditions: 5 cycles at 94°C for 30 s and 72°C for 3 min followed by 5 cycles at 94°C for 30 s, 70°C for 30 s, and 72°C for 3 min, and finally 25 cycles at 94°C for 30 s, 68°C for 30 s, and 72°C for 3 min. All PCR products for 5ʹ and 3ʹ-RACE were then mixed separately and applied to nested PCR analyses using SrPKR2 for 5ʹ-RACE and SrPKF2 for 3ʹ-RACE with Nested Universal Primer A (NUP), respectively. PCR amplification was performed under the following conditions: 1 cycle at 94°C for 2 min, followed by 25 cycles of 94°C for 30 s, 68°C for 30 s, and 72°C for 1 min, and a final extension at 72°C for 7 min.

SrPEPtaseF2 and SrPEPtaseR2 primers were used with UPM to amplify the gene for *SrPEPtase* in 3ʹ- and 5ʹ-RACE analyses, respectively. PCR reactions were performed under the following conditions: 5 cycles at 94°C for 30 s and 72°C for 3 min, followed by 5 cycles at 94°C for 30 s, 70°C for 30 s, and 72°C for 3 min, and, finally, 25 cycles at 94°C for 30 s, 60°C for 30 s, and 72°C for 3 min. PCR products of 3ʹ- and 5ʹ-RACE were used with NUP for nested PCR using SrPEPtaseF3 for 3ʹ-RACE and SrPEPtaseR2 for 5ʹ-RACE. PCR amplification was performed under the following conditions: 1 cycle at 94°C for 2 min, followed by 25 cycles of 94°C for 30 s, 60°C for 30 s, and 72°C for 1 min, and a final extension at 72°C for 7 min.

SrPEPCF2 and SrPEPCR2 were used for 3ʹ- and 5ʹ-RACE of *SrPEPC*, respectively. PCR amplification was performed under the following conditions: 5 cycles at 94°C for 30 s and 72°C for 3 min, followed by 5 cycles at 94°C for 30 s, 70°C for 30 s, and 72°C for 3 min, and, finally, 25 cycles at 94°C for 30 s, 68°C for 30 s, and 72°C for 3 min.

SrPEPCKF2 and SrPEPCKR2 were used with UPM to amplify for 3ʹ- and 5ʹ-RACE of *SrPEPCK*, respectively, followed by nested PCR using SrPEPCKR2 and SrPEPCKF3 with NUP for 5ʹ- and 3ʹ-RACE of *SrPEPCK*, respectively. PCR amplification was performed under the following conditions: 5 cycles at 94°C for 30 s and 72°C for 3 min, followed by 5 cycles at 94°C for 30 s, 70°C for 30 s and 72°C for 3 min, and finally 25 cycles at 94°C for 30 s, 68°C for 30 s and 72°C for 3 min. The PCR products of 3ʹ- and 5ʹ-RACE were used for nested PCR using SrPEPCKF3 for 3ʹ-RACE and SrPEPCKR2 for 5ʹ-RACE with NUP. PCR amplification was performed under the following conditions: 1 cycle at 94°C for 2 min, followed by 25 cycles of 94°C for 30 s, 68°C for 30 s and 72°C for 1 min with a final extension at 72°C for 7 min.

PCR products of 3ʹ- and 5ʹ-RACE of *SrPK*, *SrPEPtase*, *SrPEPC* and *SrPEPCK* were purified by QuickStep^TM^2 PCR Purification Kit (Edge Bio) and then cloned into pMD19 with DNA Ligation Kit <Mighty Mix>. After transformation into *E.coli*, at least 10 colonies of 3ʹ- RACE and 5ʹ-RACE of all these genes were identified by colony PCR, and each plasmid was purified by LaboPass^TM^ Mini Plasmid DNA Purification Kit for sequencing.

To isolate full-length cDNAs, final PCR amplifications were performed with KOD polymerase (KOD -Plus-, Toyobo) using the following gene-specific primers: SrPKF5-SrPKR5 for *SrPK*; SrPEPCF3-SrPEPCR3 for *SrPEPC*, and SrPEPCKF4-SrPEPCKR3 for *SrPEPCK*. PCR amplification was performed under the following conditions: 1 cycle at 94°C for 2 min, followed by 35 cycles at 94°C for 2 min, 55°C for 30 s, and 68°C for 2 min. The primers SrPEPtaseF4-SrPEPtaseR4 for *SrPEPtase* were used under the following PCR conditions: 1 cycle at 94°C for 2 min, followed by 35 cycles at 94°C for 15 s, 60°C for 30 s, and 68°C for 2 min. Amplified PCR products were then subjected to 10x A-attachment mix (Toyobo), and incubated at 60°C for 10 min. Products were purified using a QuickStep^TM^2 PCR Purification Kit. Purified fragments were then cloned into pMD19 using DNA Ligation Kit <Mighty Mix>. After transformation into *E.coli*, colonies of *SrPK*, *SrPEPtase*, *SrPEPC* and *SrPEPCK* were identified by colony PCR, and each plasmid was purified by LaboPass^TM^ Mini Plasmid DNA Purification Kit for sequencing in both directions. Full-length sequences were determined by isolating at least four clones, including at least two clones with identical inserts.
