# Supplementary material for: Metabolic interplay between cytosolic phosphoenolpyruvate carboxylase and mitochondrial alternative oxidase in thermogenic skunk cabbage, Symplocarpus renifolius
Source: Plant Signal Behav. 2016 Oct 14;11(11):e1247138. doi: 10.1080/15592324.2016.1247138 (PMC5157899; doi:10.1080/15592324.2016.1247138)
Supplement: Supplemental_data.zip [file kpsb-11-11-1247138-s001.zip › Supplemental data/2. Supplementary Fig. 1_final.pptx]

## Slide 1
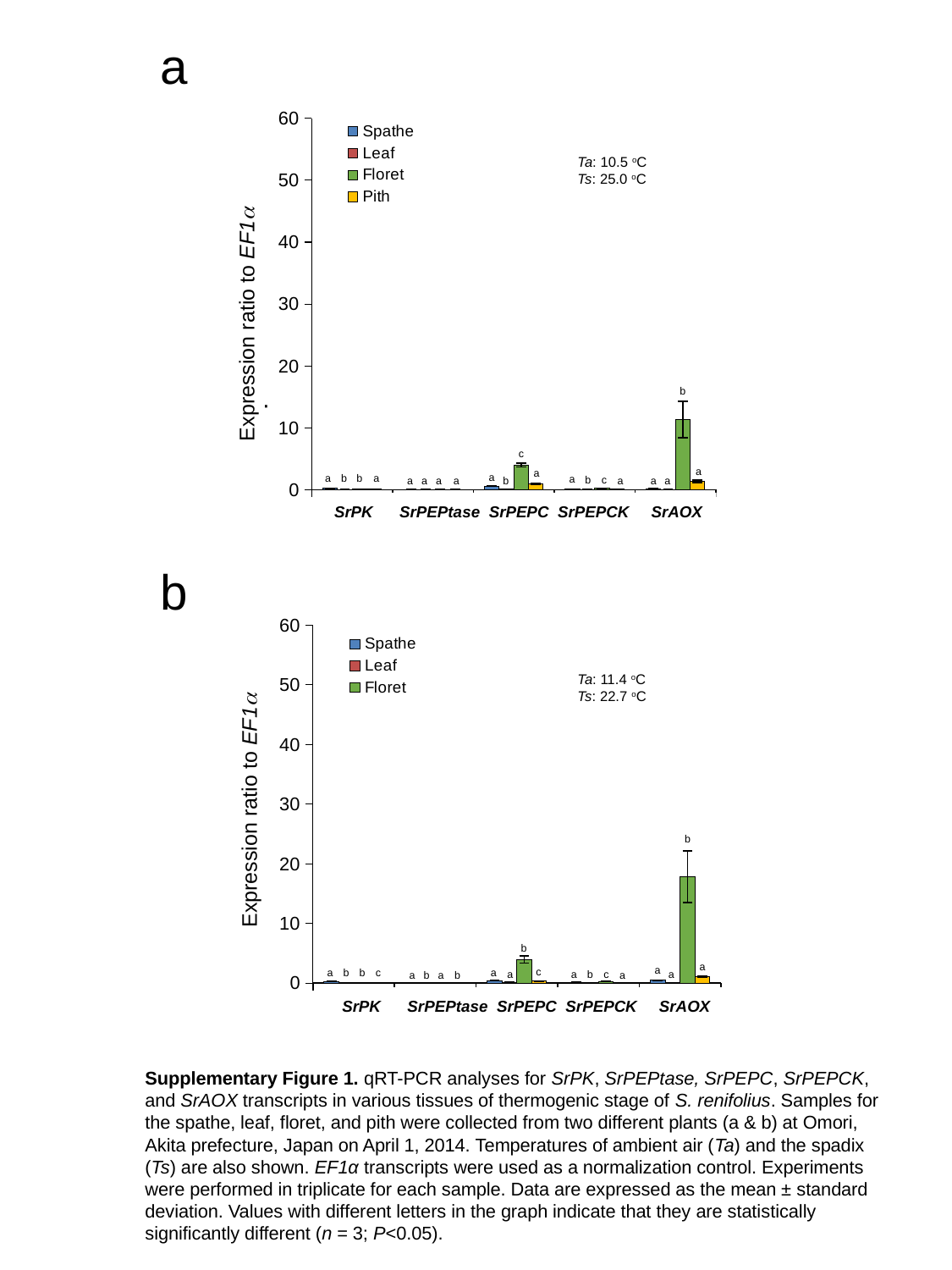

a
### Chart
| Category | Spathe | Leaf | Floret | Pith |
|---|---|---|---|---|
| SrPK | 0.179780429857751 | 0.00854462934621607 | 0.105072877481679 | 0.0540716101809308 |
| SrPEPtase | 0.00794046666993396 | 0.00115232024087015 | 0.0102575773184937 | 0.00105853273281534 |
| SrPEPC | 0.539838775339073 | 0.0576340719059699 | 3.96733264471267 | 0.920640916896057 |
| SrPEPCK | 0.0622436610435171 | 0.00998231818288593 | 0.196940955347839 | 0.0829092084967464 |
| SrAOX | 0.12689001323916 | 0.0154186526190425 | 11.32656696506944 | 1.327949702180805 |Expression ratio to EF1a
 SrPK SrPEPtase SrPEPC SrPEPCK SrAOX
b
### Chart
| Category | Spathe | Leaf | Floret | Pith |
|---|---|---|---|---|
| SrPK | 0.275452883332783 | 0.0333575439810858 | 0.0843001009743756 | 0.0824597143215592 |
| SrPEPtase | 0.0151671190196673 | 0.0023007224049621 | 0.0121262049029842 | 0.00169470007267608 |
| SrPEPC | 0.40381567503945 | 0.102158482204271 | 3.926544082384279 | 0.296138402281335 |
| SrPEPCK | 0.104722976878509 | 0.00779783296509104 | 0.246017273391755 | 0.067321061012831 |
| SrAOX | 0.456445503170977 | 0.0276990853318696 | 17.79856313475963 | 1.043984967107789 |Expression ratio to EF1a
 SrPK SrPEPtase SrPEPC SrPEPCK SrAOX
Ta: 10.5 oC
Ts: 25.0 oC
b
c
a
a
a
a
b
b
a
a
b
c
a
b
a
a
a
a
a
a
Ta: 11.4 oC
Ts: 22.7 oC
b
b
a
a
c
a
a
b
b
c
a
a
b
c
a
a
a
b
a
b
Supplementary Figure 1. qRT-PCR analyses for SrPK, SrPEPtase, SrPEPC, SrPEPCK, and SrAOX transcripts in various tissues of thermogenic stage of S. renifolius. Samples for the spathe, leaf, floret, and pith were collected from two different plants (a & b) at Omori, Akita prefecture, Japan on April 1, 2014. Temperatures of ambient air (Ta) and the spadix (Ts) are also shown. EF1α transcripts were used as a normalization control. Experiments were performed in triplicate for each sample. Data are expressed as the mean ± standard deviation. Values with different letters in the graph indicate that they are statistically significantly different (n = 3; P<0.05).
