# Supplementary material for: Metabolic interplay between cytosolic phosphoenolpyruvate carboxylase and mitochondrial alternative oxidase in thermogenic skunk cabbage, Symplocarpus renifolius
Source: Plant Signal Behav. 2016 Oct 14;11(11):e1247138. doi: 10.1080/15592324.2016.1247138 (PMC5157899; doi:10.1080/15592324.2016.1247138)
Supplement: Supplemental_data.zip [file kpsb-11-11-1247138-s001.zip › Supplemental data/4. Supplementary Table 1_final.pptx]

## Slide 1
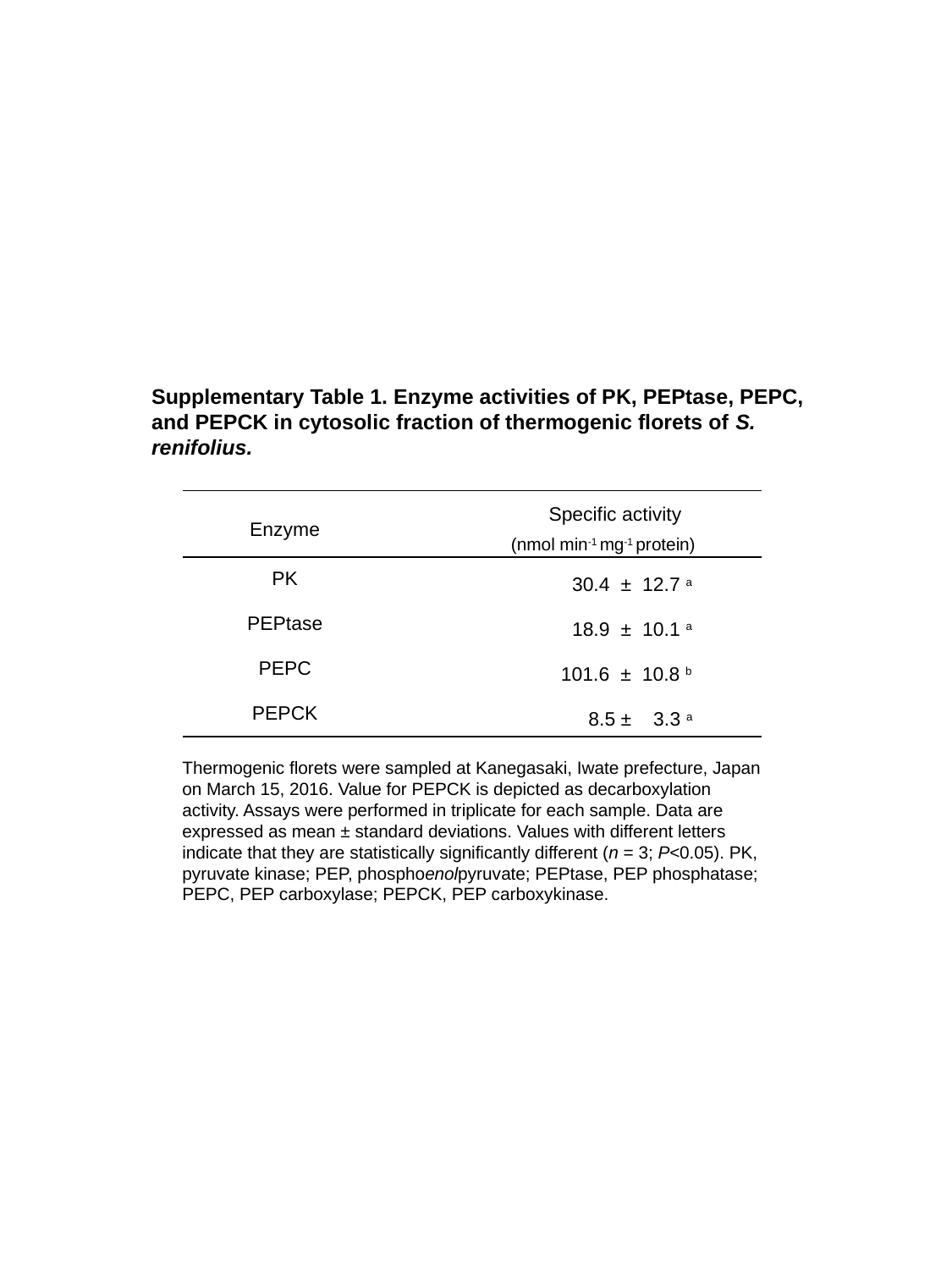

Supplementary Table 1. Enzyme activities of PK, PEPtase, PEPC, and PEPCK in cytosolic fraction of thermogenic florets of S. renifolius.
| Enzyme | Specific activity (nmol min-1 mg-1 protein) |
| --- | --- |
| PK | 30.4 ± 12.7 a |
| PEPtase | 18.9 ± 10.1 a |
| PEPC | 101.6 ± 10.8 b |
| PEPCK | 8.5 ± 3.3 a |
Thermogenic florets were sampled at Kanegasaki, Iwate prefecture, Japan on March 15, 2016. Value for PEPCK is depicted as decarboxylation activity. Assays were performed in triplicate for each sample. Data are expressed as mean ± standard deviations. Values with different letters indicate that they are statistically significantly different (n = 3; P<0.05). PK, pyruvate kinase; PEP, phosphoenolpyruvate; PEPtase, PEP phosphatase; PEPC, PEP carboxylase; PEPCK, PEP carboxykinase.
