# Supplementary material for: Metabolic interplay between cytosolic phosphoenolpyruvate carboxylase and mitochondrial alternative oxidase in thermogenic skunk cabbage, Symplocarpus renifolius
Source: Plant Signal Behav. 2016 Oct 14;11(11):e1247138. doi: 10.1080/15592324.2016.1247138 (PMC5157899; doi:10.1080/15592324.2016.1247138)
Supplement: Supplemental_data.zip [file kpsb-11-11-1247138-s001.zip › Supplemental data/5. Supplementary Table 2_final.pptx]

## Slide 1
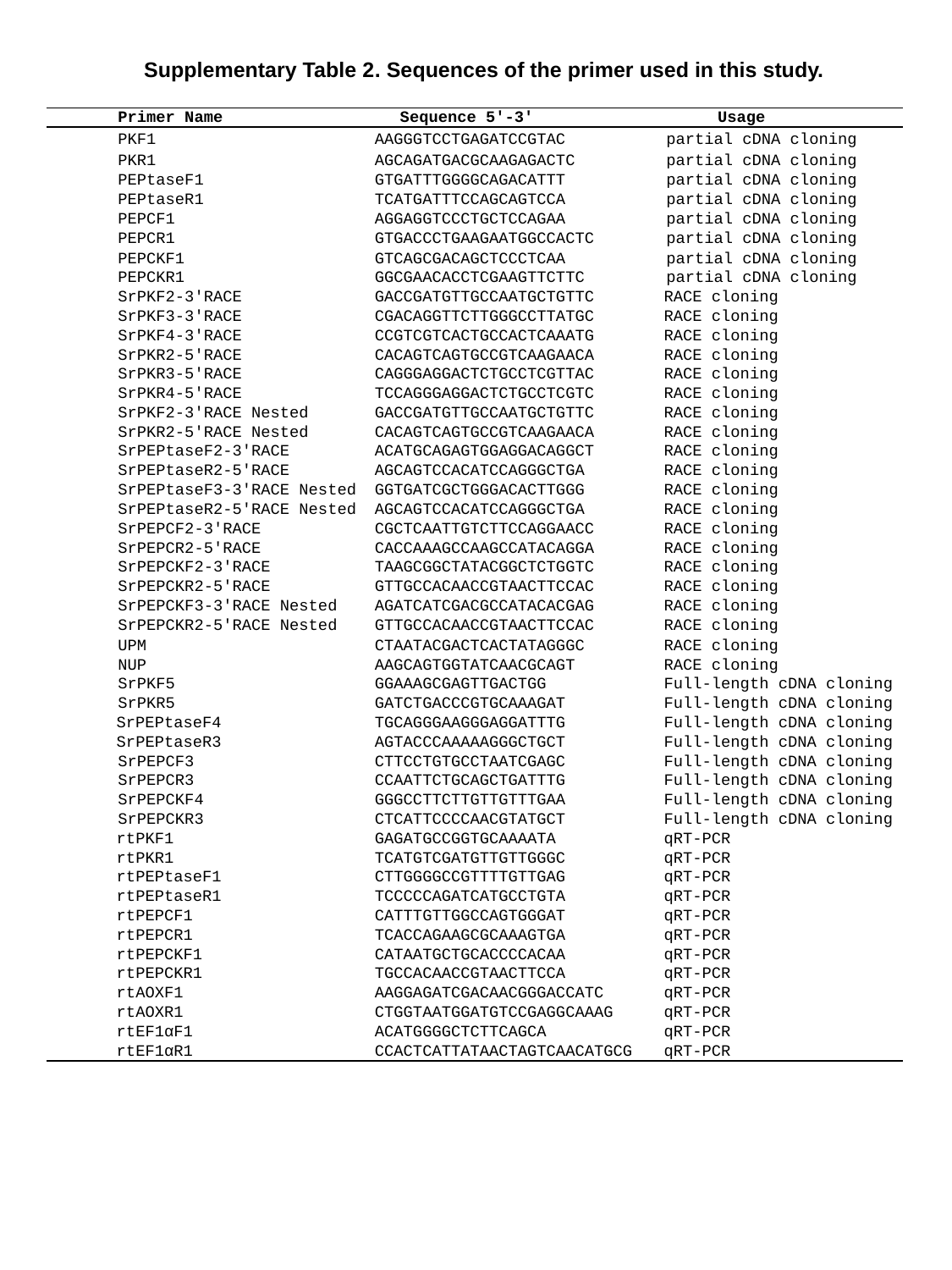

Supplementary Table 2. Sequences of the primer used in this study.
| Primer Name | Sequence 5ʹ-3ʹ | Usage |
| --- | --- | --- |
| PKF1 | AAGGGTCCTGAGATCCGTAC | partial cDNA cloning |
| PKR1 | AGCAGATGACGCAAGAGACTC | partial cDNA cloning |
| PEPtaseF1 | GTGATTTGGGGCAGACATTT | partial cDNA cloning |
| PEPtaseR1 | TCATGATTTCCAGCAGTCCA | partial cDNA cloning |
| PEPCF1 | AGGAGGTCCCTGCTCCAGAA | partial cDNA cloning |
| PEPCR1 | GTGACCCTGAAGAATGGCCACTC | partial cDNA cloning |
| PEPCKF1 | GTCAGCGACAGCTCCCTCAA | partial cDNA cloning |
| PEPCKR1 | GGCGAACACCTCGAAGTTCTTC | partial cDNA cloning |
| SrPKF2-3′RACE | GACCGATGTTGCCAATGCTGTTC | RACE cloning |
| SrPKF3-3′RACE | CGACAGGTTCTTGGGCCTTATGC | RACE cloning |
| SrPKF4-3′RACE | CCGTCGTCACTGCCACTCAAATG | RACE cloning |
| SrPKR2-5′RACE | CACAGTCAGTGCCGTCAAGAACA | RACE cloning |
| SrPKR3-5′RACE | CAGGGAGGACTCTGCCTCGTTAC | RACE cloning |
| SrPKR4-5′RACE | TCCAGGGAGGACTCTGCCTCGTC | RACE cloning |
| SrPKF2-3′RACE Nested | GACCGATGTTGCCAATGCTGTTC | RACE cloning |
| SrPKR2-5′RACE Nested | CACAGTCAGTGCCGTCAAGAACA | RACE cloning |
| SrPEPtaseF2-3′RACE | ACATGCAGAGTGGAGGACAGGCT | RACE cloning |
| SrPEPtaseR2-5′RACE | AGCAGTCCACATCCAGGGCTGA | RACE cloning |
| SrPEPtaseF3-3′RACE Nested | GGTGATCGCTGGGACACTTGGG | RACE cloning |
| SrPEPtaseR2-5′RACE Nested | AGCAGTCCACATCCAGGGCTGA | RACE cloning |
| SrPEPCF2-3′RACE | CGCTCAATTGTCTTCCAGGAACC | RACE cloning |
| SrPEPCR2-5′RACE | CACCAAAGCCAAGCCATACAGGA | RACE cloning |
| SrPEPCKF2-3′RACE | TAAGCGGCTATACGGCTCTGGTC | RACE cloning |
| SrPEPCKR2-5′RACE | GTTGCCACAACCGTAACTTCCAC | RACE cloning |
| SrPEPCKF3-3′RACE Nested | AGATCATCGACGCCATACACGAG | RACE cloning |
| SrPEPCKR2-5′RACE Nested | GTTGCCACAACCGTAACTTCCAC | RACE cloning |
| UPM | CTAATACGACTCACTATAGGGC | RACE cloning |
| NUP | AAGCAGTGGTATCAACGCAGT | RACE cloning |
| SrPKF5 | GGAAAGCGAGTTGACTGG | Full-length cDNA cloning |
| SrPKR5 | GATCTGACCCGTGCAAAGAT | Full-length cDNA cloning |
| SrPEPtaseF4 | TGCAGGGAAGGGAGGATTTG | Full-length cDNA cloning |
| SrPEPtaseR3 | AGTACCCAAAAAGGGCTGCT | Full-length cDNA cloning |
| SrPEPCF3 | CTTCCTGTGCCTAATCGAGC | Full-length cDNA cloning |
| SrPEPCR3 | CCAATTCTGCAGCTGATTTG | Full-length cDNA cloning |
| SrPEPCKF4 | GGGCCTTCTTGTTGTTTGAA | Full-length cDNA cloning |
| SrPEPCKR3 | CTCATTCCCCAACGTATGCT | Full-length cDNA cloning |
| rtPKF1 | GAGATGCCGGTGCAAAATA | qRT-PCR |
| rtPKR1 | TCATGTCGATGTTGTTGGGC | qRT-PCR |
| rtPEPtaseF1 | CTTGGGGCCGTTTTGTTGAG | qRT-PCR |
| rtPEPtaseR1 | TCCCCCAGATCATGCCTGTA | qRT-PCR |
| rtPEPCF1 | CATTTGTTGGCCAGTGGGAT | qRT-PCR |
| rtPEPCR1 | TCACCAGAAGCGCAAAGTGA | qRT-PCR |
| rtPEPCKF1 | CATAATGCTGCACCCCACAA | qRT-PCR |
| rtPEPCKR1 | TGCCACAACCGTAACTTCCA | qRT-PCR |
| rtAOXF1 | AAGGAGATCGACAACGGGACCATC | qRT-PCR |
| rtAOXR1 | CTGGTAATGGATGTCCGAGGCAAAG | qRT-PCR |
| rtEF1αF1 | ACATGGGGCTCTTCAGCA | qRT-PCR |
| rtEF1αR1 | CCACTCATTATAACTAGTCAACATGCG | qRT-PCR |
